# Preparation and characterization of size-controlled glioma spheroids using agarose hydrogel microwells

**DOI:** 10.1371/journal.pone.0211078

**Published:** 2019-01-24

**Authors:** Fereshtehsadat Mirab, You Jung Kang, Sheereen Majd

**Affiliations:** 1 Department of Biomedical Engineering, University of Houston, Houston, Texas, United States of America; 2 Department of Biomedical Engineering, Pennsylvania State University, University Park, Pennsylvania, United States of America; Monash University, AUSTRALIA

## Abstract

Treatment of glioblastoma, the most common and aggressive type of primary brain tumors, is a major medical challenge and the development of new alternatives requires simple yet realistic models for these tumors. *In vitro* spheroid models offer attractive platforms to mimic the tumor behavior *in vivo* and have thus, been increasingly applied for assessment of drug efficacy in various tumors. The aim of this study was to produce and characterize size-controlled U251 glioma spheroids towards application in glioma drug evaluation studies. To this end, we fabricated agarose hydrogel microwells with cylindrical shape and diameters of 70–700 μm and applied these wells without any surface modification for glioma spheroid formation. The resultant spheroids were homogeneous in size and shape, exhibited high cell viability (> 90%), and had a similar growth rate to that of natural brain tumors. The final size of spheroids depended on cell seeding density and microwell size. The spheroids’ volume increased linearly with the cell seeding density and the rate of this change increased with the well size. Lastly, we tested the therapeutic effect of an anti-cancer drug, Di-2-pyridylketone-4,4-dimethyl-3-thiosemicarbazone (Dp44mT) on the resultant glioma spheroids and demonstrated the applicability of this spheroid model for drug efficacy studies.

## 1. Introduction

Glioblastoma multiforme (GBM, grade IV glioma) is the most common and aggressive type of primary brain tumor and has the worst prognosis among these tumors [1]. Traditional treatment strategies that include surgery, radiation therapy, and chemotherapy have limited success in eliminating these tumors, often leading to their fatal recurrence [2, 3]. Development of more effective treatments for this deadly cancer requires simple yet realistic tumor models that enable proper evaluation of their therapeutic efficacy prior to the clinical use. For several decades, drug efficacy evaluations have been performed on two-dimensional (2D) *in vitro* disease models formed on flat and rigid substrates. Despite the ease of use, these 2D cultures often fail to predict the *in vivo* tissue response to candidate drugs as they do not resemble the natural tumor stromal heterogeneity, cell-cell interactions, gradient in nutrient concentrations, and extracellular matrix (ECM) compounds [4]. Moreover, culturing cells in a monolayer format leads to a default apical-basal polarity that is different from the spatial organization of cells in natural tissues, making cells more sensitive to anticancer drugs [5]. Recent efforts have thus, been focused on the development of more physiologically relevant models that can better predict the response of natural tumors to candidate anti-cancer compounds [4].

Multicellular tumor spheroids (i.e. self-assembled spherical clusters of tumor cells) have emerged as promising *in vitro* three-dimensional (3D) models that can recapitulate many functional and structural properties of solid tumors *in vivo* [6]. In addition, their ease of assembly, reproducibility, and capability to provide high-throughput drug screening has rendered them as one of the most popular models for preclinical evaluation of therapeutic compounds [4, 6]. Multicellular spheroids can be prepared via a number of techniques including hanging drops [7], spinner flask (or rotary) cultures [8], overlay on non-adhesive surfaces [9], and microwells [10, 11]. Among these techniques, the use of hydrogel microwells has proven to be particularly suitable for controlling the size of spheroids while maintaining high cell viability during/after spheroid formation due to the ECM-mimicking biophysical properties of hydrogels [6, 12, 13]. These microwell platforms have been prepared using variety of gels such as agarose [12, 14], chitosan [15], poly(ethylene glycol) (PEG) [11, 13], and gelatin methacrylate (GelMA) [16]. Among these hydrogels, agarose is particularly attractive as it is natural, low-cost, non-toxic, and cell-repellent, which induces spheroid formation and inhibits random cell adhesion. Moreover, agarose permeability to gas and small biomolecules allows for diffusion of nutrients and drugs, which is critical for spheroid studies [15, 17].

Hydrogel microwells have been utilized to prepare 3D tissue models using a number of cells including hepatoblastoma (HepG2) [18] and embryonic [19] stem cells, fibroblasts [14], and cancer cells [11, 16]. However, only a few studies have investigated the formation of glioma spheroids via hydrogel microwells [11, 14]. Particularly, no previous work has focused on the preparation of spheroids from U251 glioma cells, a widely applied cell line for evaluation of anti-cancer drugs against brain tumors, using hydrogel microwells. Although an overlay method [9] has been previously utilized for the preparation of U251 glioma spheroids, the hydrogel microwell-based approach can provide better control over the size and uniformity of these spheroids, which is particularly advantageous for drug evaluation studies.

The objective of this study was to prepare size-controlled and highly viable glioma spheroids from U251 cell line using agarose microwells and to evaluate their potential for therapeutic efficacy studies. To this end, we designed and fabricated agarose gel microwells of cylindrical shape with diameters of 70, 150, 450, and 700 μm by replica molding method and employed these wells, without any surface modification, for producing glioma spheroids. We evaluated cell viability in the resultant spheroids in comparison to monolayer cultures and investigated the effect of microwell size and cell seeding density on the size of the resultant spheroids. We assessed the growth rate of spheroids in a week to compare with the reported growth rate of natural glioma tumors in the body. Finally, we examined the effect of an anti-cancer agent, Di-2- pyridylketone-4,4-dimethyl-3-thiosemicarbazone (Dp44mT), on these glioma spheroids to verify the applicability of these tumor models for drug efficacy studies. Dp44mT is a chelator with proven anti-proliferative activity in variety of cancers including lung carcinoma and melanoma [20, 21]. The ability of Dp44mT to chelate iron and cupper and redox cycle the resultant complexes, makes this compound highly cytotoxic. Moreover, Dp44mT has proven capable of overcoming multidrug-resistance, which is the major obstacle for the success of chemotherapy in cancer treatment [22, 23]. Dp44mT is thus an attractive candidate for treatment of glioma tumors.

## 2. Materials and methods

### 2.1. Materials

Agarose was acquired from OmniPur (Merck, Darmstadt, Germany). SYLGARD 184 silicone elastomer kit, including poly(dimethylsiloxane) (PDMS) base and catalyst, was purchased from Dow Corning (Auburn, MI). Di-2-pyridylketone-4,4-dimethyl-3- thiosemicarbazone (Dp44mT), ribonuclease A (RNase A), and propidium iodide (PI) were purchased from Sigma-Aldrich (St. Louis, MO). Dulbecco’s modified Eagle’s medium (DMEM), Dulbecoco’s phosphate-buffered saline (DPBS), fetal bovine serum (FBS), and penicillin-streptomycin mixtures (antibiotics) were from Gibco BRL (Carlsbad, CA). Glioma cell line U251 was from ATCC (Manassas, VA). SYTO 10 and ethidium homodimer-2 (EthD-2) were purchased from Invitrogen (Waltham, MA).

### 2.2. Preparation of hydrogel microwells

**Fig 1:**
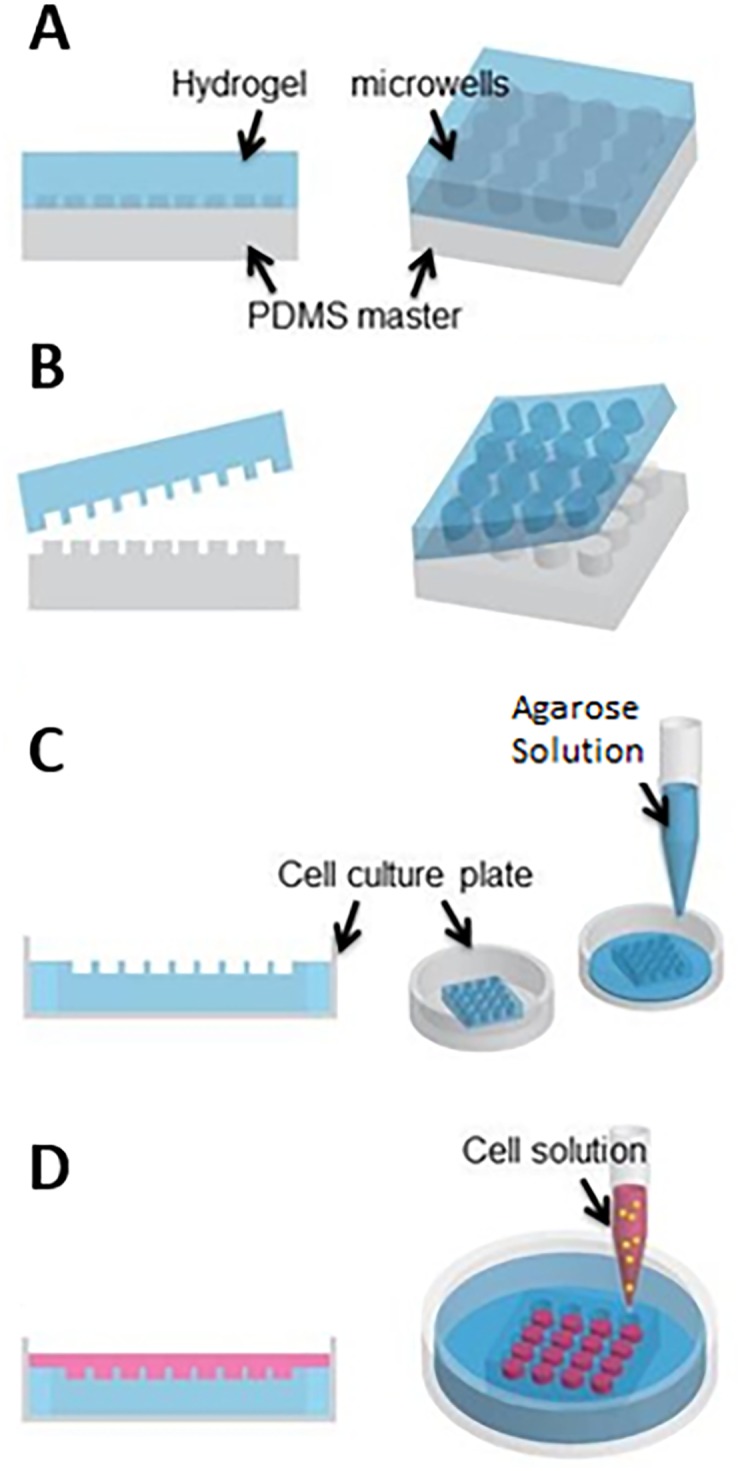
Schematic of agarose microwell fabrication and cell seeding for preparation of glioma spheroids.

Agarose hydrogel microwells were fabricated by replica-molding method [24, 25] as depicted in Fig 1A–1C. In brief, we designed and fabricated PDMS masters with positive circular posts of 70, 150, 450, and 700 μm diameter. An aqueous solution of 2.5% (w/v) agarose was poured onto the desired PDMS master (Fig 1A) followed by degassing to remove the air bubbles. Upon agarose gelation at 4° C for about 1 hr, the array of agarose microwells was unmolded (Fig 1B) and placed into a 24-well cell culture plate. The space surrounding the gel microwells within each well was filled with 1% (w/v) aqueous agarose solution (Fig 1C). After gelation of the additional agarose solution, the hydrogel microwells in the culture plate were incubated in DPBS under UV light overnight.

### 2.3. Cell culture and spheroid formation

Human malignant glioma cells U251 were cultured in DMEM supplemented with 10% FBS and 2% antibiotic at 37° C in a humid incubator with 5% CO_2_. Cells were then trypsinized and harvested in DMEM supplemented with 10% FBS and antibiotics. We counted the number of cells using a hemocytometer. The U251 cells were seeded in a 24-well culture plate at a cell density of 3.0 × 10^5^ cells/well to form the control monolayers and were also seeded on the agarose microwells (Fig 1D) at cell densities of: 4.0 × 10^4^, 1.0 × 10^5^, and 2.0 × 10^5^ cells/well for 70 μm microwells, 6.0 × 10^4^, 1.5 × 10^5^, 2.4 × 10^5^, and 4.2 × 10^5^ cells/well for 150 μm microwells, and 1.0 × 10^5^, 3.0 × 10^5^, 4.8 × 10^5^, 6.4 × 10^5^, and 10.0 × 10^5^ cells/well for 450 μm microwells. Cells began to aggregate within few hours and formed into glioma spheroids within one day. Two days after the cell seeding, the spheroids were imaged under an inverted microscope (Zeiss Axio Observer Z1 microscope, Carl Zeiss, Oberkochen, Germany). We measured the spheroid diameter and estimated their volume assuming a perfectly spherical shape.

### 2.4. Cellular viability studies

Cell viability was assessed using a LIVE/DEAD assay. U251 monolayers and spheroids were prepared as described above and incubated in cell culture medium supplemented with 50 μM SYTO 10 green (to label live cells) and 50 μM EthD-2 (to label membrane-damaged or dead cells) for 15 min at 37° C. After staining, the culture medium was replaced and cells were imaged under an inverted microscope (Zeiss Axio Observer Z1 microscope, Carl Zeiss, Oberkochen, Germany). The images were analyzed using the AxioVision 4.8.2 software (Carl Zeiss, Germany). To estimate the cell viability, the green, red, and bicolored cells were counted and used for calculating the percentage of cells with exclusively green fluorescence (i.e. viable cells).

### 2.5. Flow cytometry

To determine population of cells undergoing apoptosis, the fragmented cellular DNA was detected via sub-G1 propidium iodide (PI) assay and examined by flow cytometry. Cells in glioma monolayer were harvested and permeabilized with 75% ethanol to allow entry of PI. Note that cells in glioma spheroids were trypsinized prior to the permeabilization. Cells were then centrifuged at 2,500 rpm for 5 min to remove the ethanol and washed with DPBS, followed by incubation in DPBS supplemented with 100 μg/mL RNase A and stained with 50 μg/mL PI at room temperature for 15 min. Finally, sub-G1 population was measured by flow cytometry (FC500, Beckman Coulter, CA) and analyzed using FlowJo software (Tree Star, Inc., Ashland, OR, USA).

### 2.6. Drug efficacy studies

To investigate the applicability of U251 glioma spheroids for drug evaluation studies, we tested the effect of a highly potent anti-tumor chelator, Dp44mT [20] on these glioma tumor models. For these experiments, we prepared U251 spheroids in 150 μm microwells and incubated them in culture medium containing 100 nM Dp44mT for five days in an incubator at 37° C. Spheroids incubated without Dp44mT under the similar conditions were used as the control. The change in size of spheroids before and after the incubation was monitored using an inverted microscope. In addition, hypodiploid sub-G1 cell populations were examined using flow cytometer as described in the previous section.

### 2.7. Statistical analysis

Student’s t-test was applied to compare experimental groups and to evaluate statistical significance.

## 3. Results and discussion

### 3.1. Preparation of size-controlled glioma spheroids in agarose microwells

**Fig 2:**
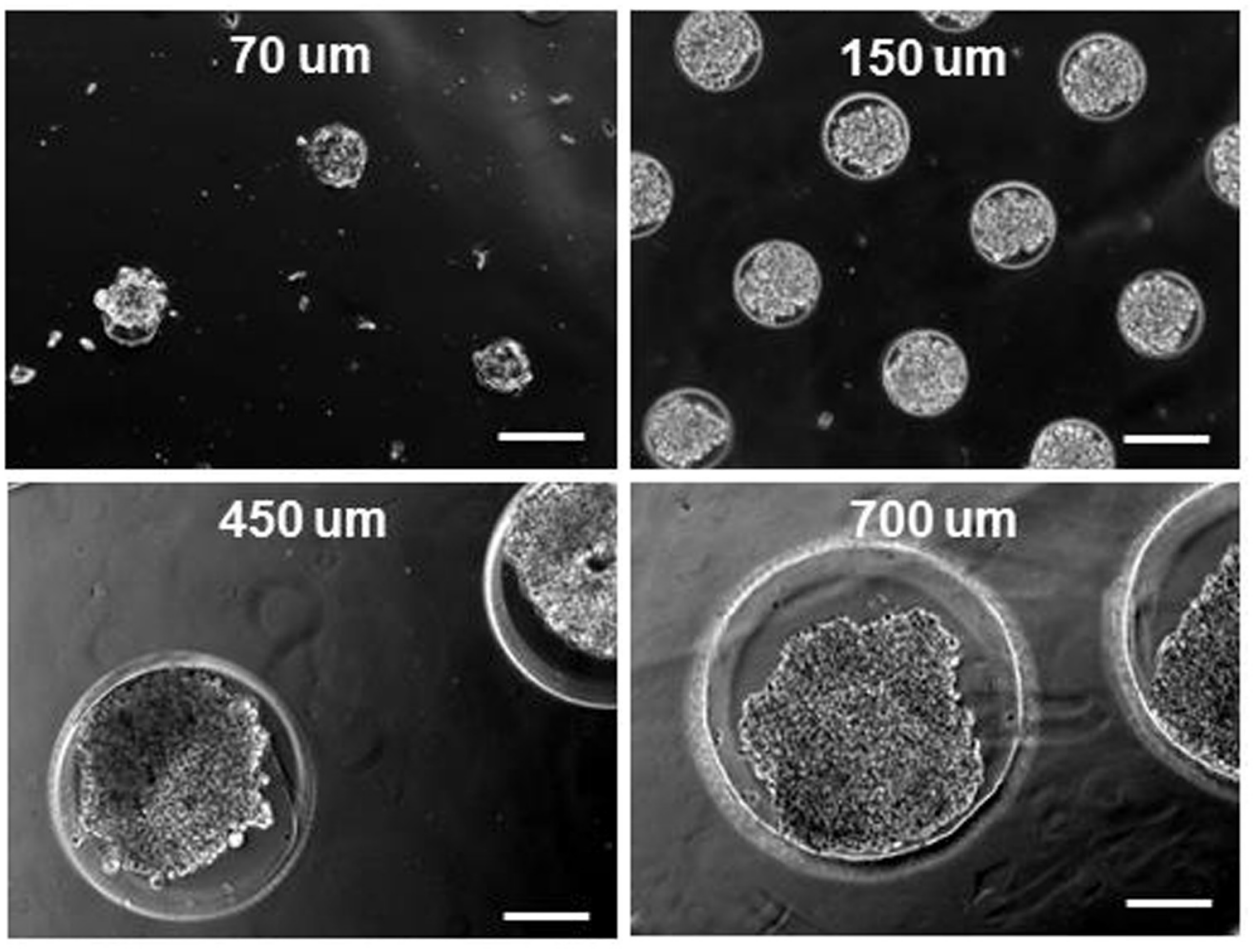
Phase contrast images of representative U251 glioma spheroids prepared in microwells of 70, 150, 450, and 700 μm diameter. Scale bars represent 200 μm.

To prepare glioma spheroids with uniform size and morphology, we seeded U251 glioma cells onto agarose microwells fabricated by soft lithography (Fig 1) with no further surface treatment. Due to the cell-repellent nature of agarose gel [26], cells began to aggregate at the bottom of microwells and self-assembled into a single spheroid within each well (Fig 2). During the spheroid formation, cells first form an aggregate and then start to secrete ECM molecules, which results in further packing of the aggregate. This packing process, also known as a spheroid compaction, typically takes between 1 to 14 days after the cell seeding depending on the technique employed for spheroid formation as well as the cell type [10, 27, 28]. In this study, formation of U251 glioma spheroids occurred within one day after the cell seeding onto hydrogel microwells, which is comparable with the reported timeline for spheroid formation from other cancer cell types using a similar approach [10]. In addition, as depicted in Fig 2, the size of the microwells enabled us to control the size of resultant spheroids. Notably, in the case of 700 μm microwells, we frequently observed formation of multiple aggregates within the individual wells (see S1 Fig). Given that the focus of this study was to produce size-controlled single spheroids per well, we did not further pursue the spheroid formation in 700 μm microwells.

### 3.2. Characterization of U251 spheroids and monolayers

**Fig 3:**
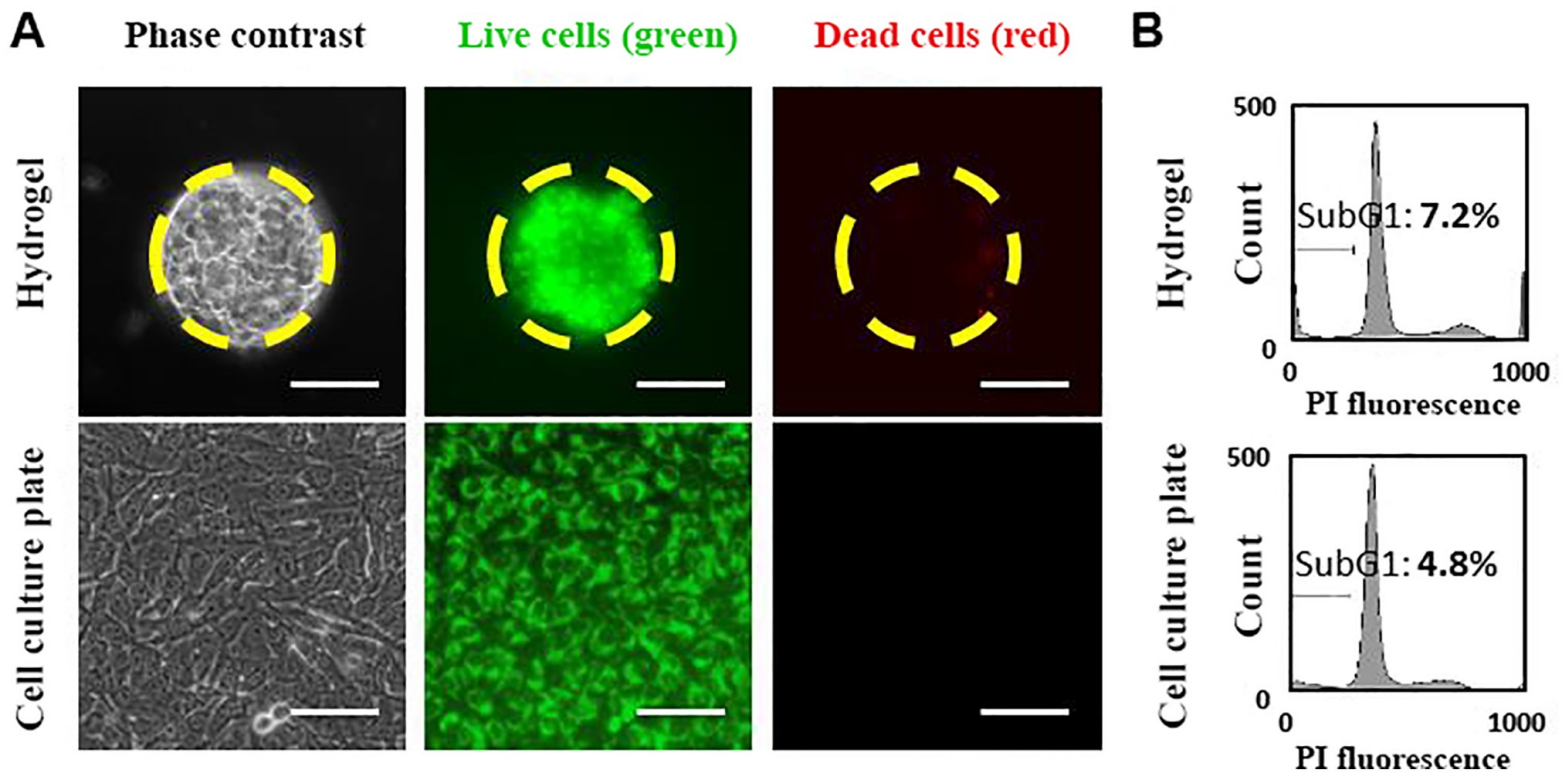
Characterization of U251 cells in spheroid and monolayer cultures. A) Phase contrast and fluorescence images of U251 cells in the form of a spheroid produced in a 150 μm well (top row) and monolayer (bottom row). Green fluorescence represents the live cells and red fluorescence represents dead cells. Yellow dashed circles mark the border of a single hydrogel microwell. Scale bars represent 80 μm. B) Sub-G1 population of U251 glioma cells in spheroids (top) and monolayers (bottom).

We examined the viability of U251 cells in both spheroid and monolayer cultures using the LIVE/DEAD assay. For these experiments, the U251 spheroids produced in 150 μm microwells and the monolayer cultures were co-labeled with SYTO 10 and EthD-2 and examined for their fluorescence signal using epifluorescence microscopy. The results showed that U251 cells in spheroids were highly viable as evident by the green fluorescence signal in Fig 3A (top row). The viability of these cells was indeed comparable to those in the monolayer cultures (Fig 3A, bottom row). We further compared the population of cells undergoing apoptosis (i.e. hypodipoloid sub-G1 cell populations) in spheroid and monolayer cultures using flow cytometry. As depicted in Fig 3B, flow cytometry data revealed that U251 cells in spheroids had a low level (~7%) of sub-G1 population, which was comparable to that in monolayer cultures (~5%). These results demonstrated the suitability of agarose microwells for production of highly viable U251 glioma spheroids.

### 3.3. Effect of microwell size and cell seeding density on U251 glioma spheroids

**Fig 4:**
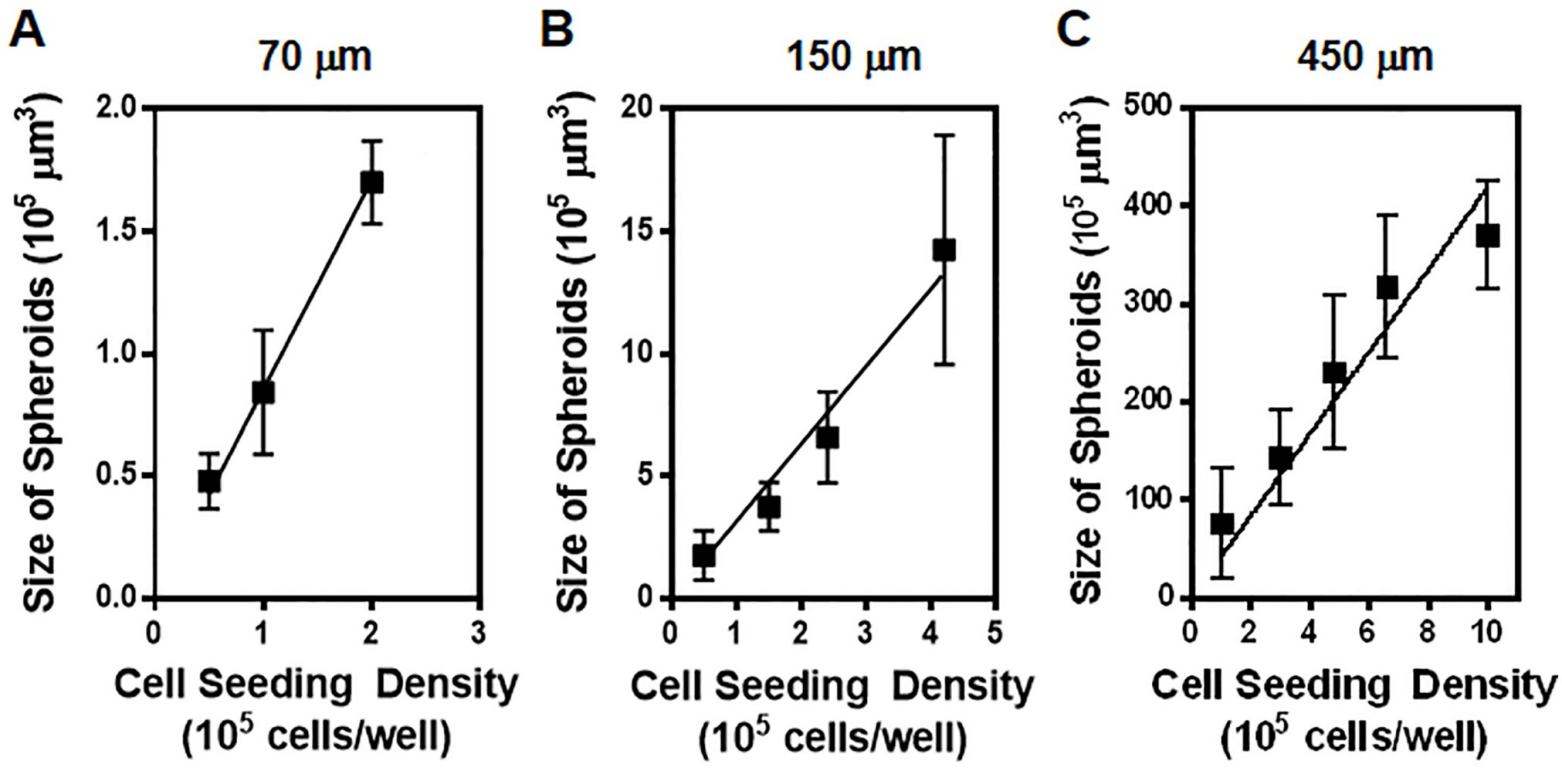
Effect of initial cell seeding density and hydrogel microwell size on the final size of U251 glioma spheroids. Graphs show the volume of U251 spheroids, measured after 2 days in culture as a function of cell seeding density per well of the culture plate that contained agarose microwells with diameters of 70 μm (A), 150 μm (B), and 450 μm (C). Data points represent the mean of 10 independent experiments and error bars represent standard deviation (SD). Solid lines show the linear fit to the data.

Size of a tumor spheroid is a critical factor that affects the transport of nutrients and therapeutics through the tumor model and can lead to inconsistent results in drug efficacy studies [29, 30]. Therefore, the spheroid size should ideally be controlled for application in drug evaluation studies. Herein, to achieve production of size-controlled U251 glioma spheroids, we examined the effect of cell seeding density and microwell size on the volume of glioma spheroids. To this end, U251 cells with various cell densities ranging from 4.0 × 10^4^ cells/well to 10.0 × 10^5^ cells/well were seeded onto agarose gel microwells with diameters of 70, 150, and 450 μm. Diameter of the resultant spheroids were measured after two days and their volumes were calculated assuming they had a spherical shape. As depicted in Fig 4, the spheroid volume increased linearly with increasing cell seeding density in all three examined well sizes. Such a positive linear correlation between the tumor size and cell seeding density has also been reported for spheroids of other cancer cell types including U87 glioma cells [11, 14] as well as cardiac side population cells (CSPCs) [31]. Moreover, these experiments revealed that the rate of this linear change (i.e. slope of fitted lines in Fig 4) increased significantly with an increase in the microwell size. Specifically, the rate of tumor size change as a function of cell density (μm^3^/cells/well) was 0.8 for 70 μm wells, 3.4 for 150 μm wells, and 43.5 for 450 μm wells. This result indicated that spheroid size becomes much more sensitive to the cell density as the well size increases, providing an excellent tool to fine-tune the tumor size.

### 3.4. Growth rate of U251 spheroids

**Fig 5:**
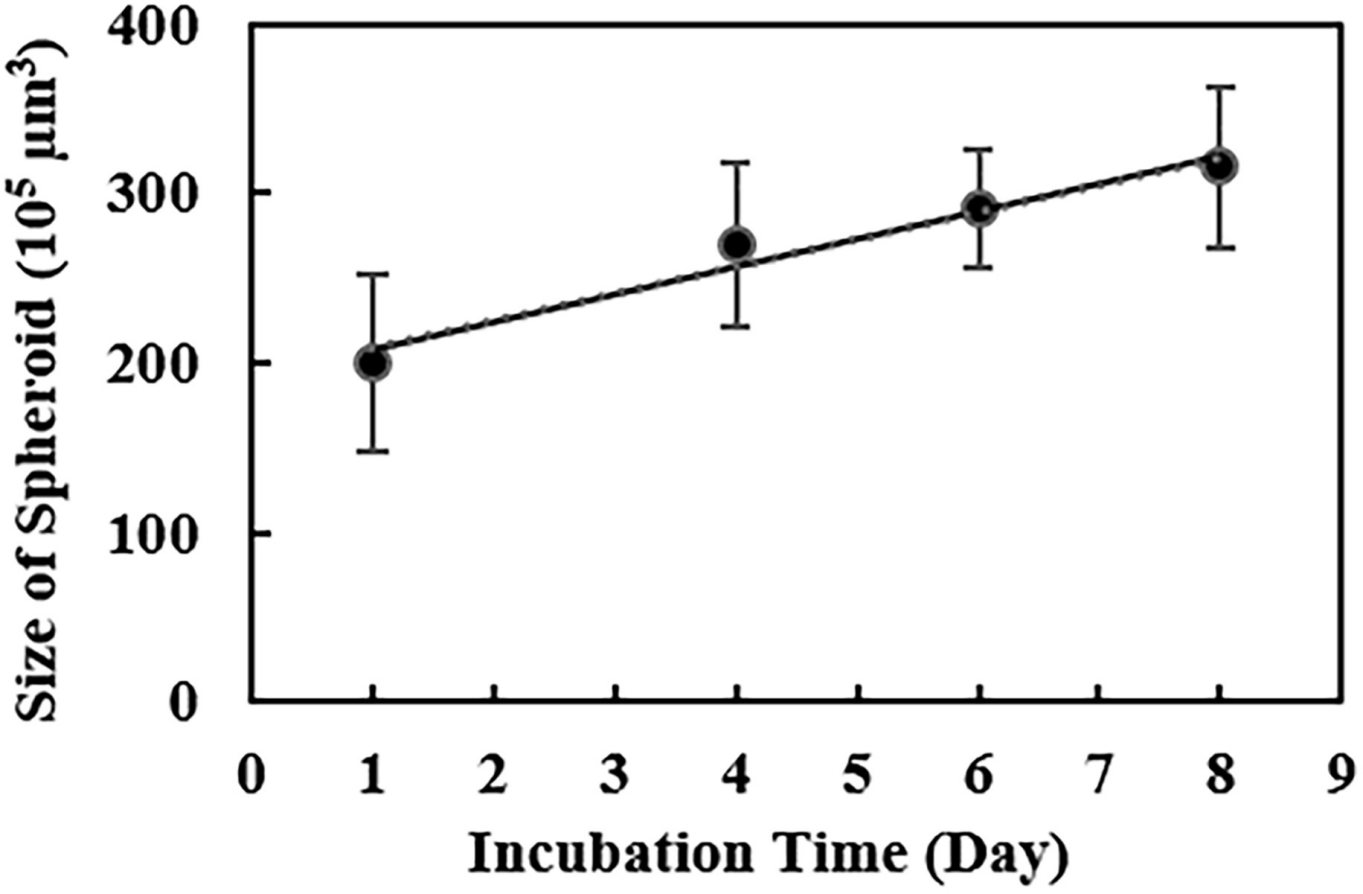
Growth rate of U251 spheroids in agarose microwells. Graph shows the measured volume of U251 spheroids as a function of time in 450 μm-sized microwells (cell seeding densities of 6.0 × 10^5^ cells/well). Data points represent the mean of 6 independent experiments and error bars show SD. Solid line shows the linear fit to data (y = 16.2x + 191.7, R^2^ = 0.9610).

In order to investigate the growth rate of spheroids in agarose microwells, we first seeded U251 cells at a seeding density of 6.0 × 10^5^ cells/well in 450 μm hydrogel microwells. For these experiments, 450 μm wells were used as these wells provided enough space for the growth of spheroids within a week. Upon formation of spheroids within one day, we monitored the diameter of spheroids using an inverted microscope for several days to estimate the tumor volume, using a previously reported approach [32]. As illustrated in Fig 5, U251 spheroids showed a gradual and linear growth of ~16% volume increase in the period of one week. Considering the linear form of this growth, the average spheroid growth rate per day was about 2.3% volume increase that is comparable to 2.2% volume increase per day found in natural glioblastoma tumors in the brain [33]. This result demonstrates that U251 spheroids produced in agarose microwells can effectively mimic the growth of natural brain tumors for at least one week.

### 3.5. Application of U251 spheroids for drug efficacy studies

**Fig 6:**
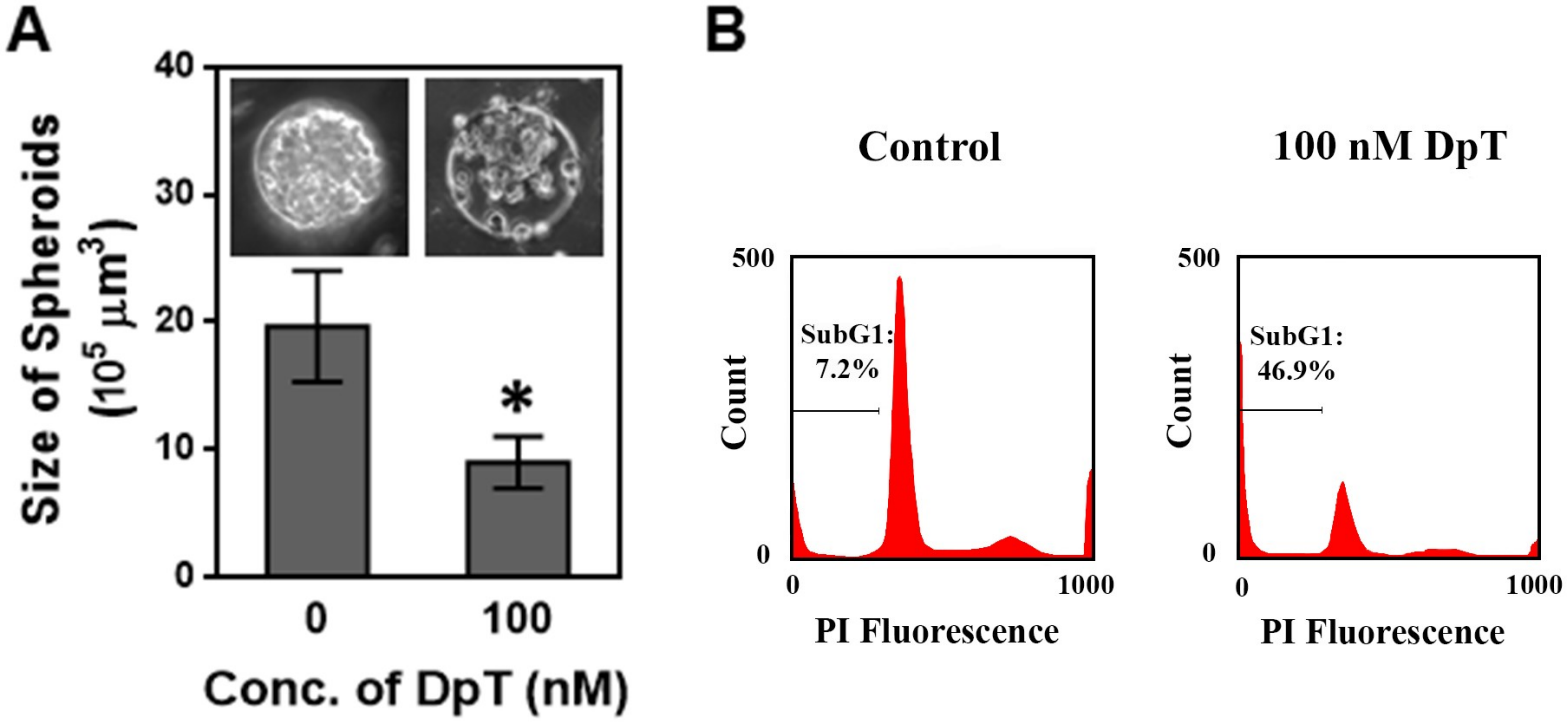
Effect of an anti-tumor agent Dp44mT on U251 spheroids. A) Bar graph shows the volume of glioma spheroids upon exposure to 0 nM Dp44mT (control) and 100 nM Dp44mT for five days. Data points represent the mean of 10 independent experiments and error bars represent SD. * represents *P* < 0.01 vs. control (0 nM). The inset shows phase contrast images of the control and Dp44mT-treated spheroids. Sale bar represents 50 μm. B) Flow cytometry data demonstrate the sub-G1 population (i.e. apoptotic cell population) in control and Dp44mT-treated (100 nM) spheroids.

To test the applicability of the produced spheroids for drug effectiveness studies, we evaluated the effect of a metal chelator with outstanding antitumor activity, Dp44mT [20], on these U251 spheroids. For this study, we prepared U251 glioma spheroids in 150 μm wells and incubated them in culture medium containing 0 nM (control) or 100 nM Dp44mT for five days. It should be noted that the well size of 150 μm was selected for these experiments as this well size produced relatively large spheroids while the resultant spheroids were also more uniform compared to those produced in 450 μm wells. Changes in the size of spheroids were monitored using an inverted microscope. As depicted in Fig 6A, we observed ~57% size reduction (corresponding to ~11.4% per day) in the spheroids treated with 100 nM Dp44mT compared to the control spheroids. This result is comparable with the previously reported results that Dp44mT treatments led to ~8% tumor size reduction per day in *in vivo* tumor models of lung carcinoma, neuroepithelioma, and neuroepithelioma [20, 23].

In addition, prior studies have shown that Dp44mT induces apoptosis in neuroepithelioma, lung carcinoma, breast cancer, and leukemic cells [23, 34]. To test the Dp44mT-induced apoptosis in gliomas, we examined hypodiploid sub-G1 populations in Dp44mT treated cells by flow cytometry as described in the literature [35]. As shown in Fig 6B, this flow cytometric analysis revealed that the apoptotic population in Dp44mT treated cells was indeed significantly higher (46.9%) than the control group (7.2%). This finding further supports our results from the size changes in Dp44mT treated spheroids and demonstrates that Dp44mT has a great level of anti-tumor activity in glioma spheroids. These results together demonstrate the potential application of these U251 spheroids for drug effectiveness studies.

## 4. Conclusion

This study describes the preparation and characterization of size-controlled glioma spheroids from a commonly used glioma cell line, U251. Agarose gel microwells with no surface treatment were applied to produce highly viable (> 90%) tumor spheroids. We demonstrated that the design of microwells and the initial cell seeding density provided great control over the final size of spheroids. The rate of growth in the glioma spheroids (~2.3% volume increase per day) was comparable to that reported for natural brain glioams (~2.2%) [33], demonstrating U251 spheroids produced in agarose microwells can effectively mimic the growth of natural brain tumors for at least one week. Moreover, we explored the applicability of these glioma spheroids for drug evaluation studies by testing the effect of an anti-tumor chelator Dp44mT on these spheroids. The results demonstrated that Dp44mT treatment effectively reduced the tumor size (~11.4% per day), which is comparable with prior reports on Dp44mT-induced size reduction of other tumor types in animal models (~8%) [20]. Moreover, flow cytometric analysis confirmed that Dp44mT induced significant apoptotic cell death in drug treated glioma spheroids, supporting the prior reports on the apoptosis inducing effect of Dp44mT on other tumor types [23, 34]. Together, these results demonstrate the great potential of the present glioma model for brain glioma studies particularly for drug performance evaluations. This model can thus, contribute to the development of more effective therapeutics for brain gliomas.

## Supporting information

S1 Fig Formation of multiple spheroids in large microwells. Optical image of multiple U251 spheroids formed in a representative 700 μm well.
(TIF)
