# Supplementary figures and images for: Preparation and characterization of size-controlled glioma spheroids using agarose hydrogel microwells
Source: PLoS One. 2019 Jan 24;14(1):e0211078. doi: 10.1371/journal.pone.0211078 (PMC6345430; doi:10.1371/journal.pone.0211078)

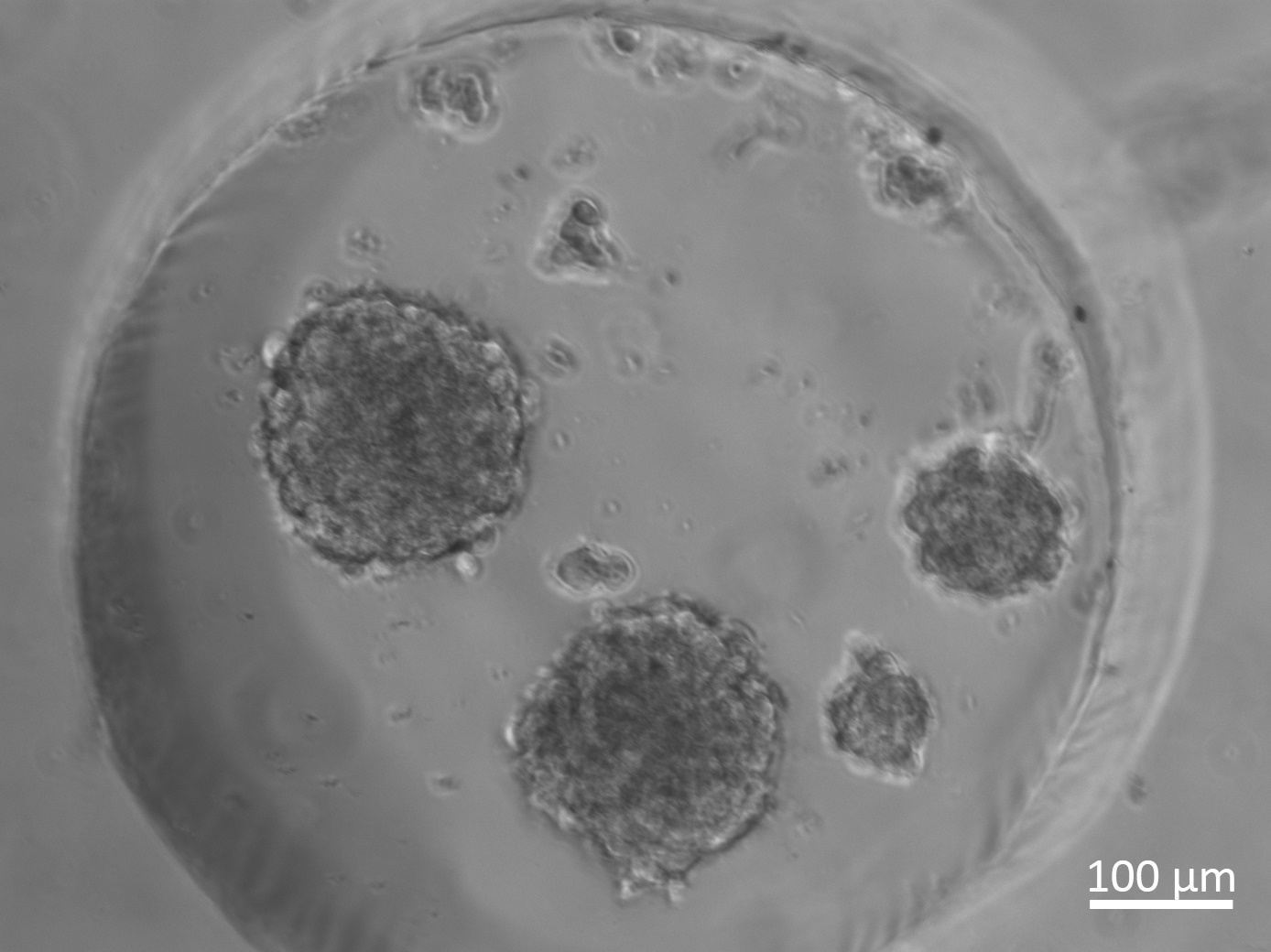

Supplement: S1 Fig — Optical image of multiple U251 spheroids formed in a representative 700 μm well. (TIF) [file pone.0211078.s001.tif]
